# Identification of Different Varieties of Sesame Oil Using Near-Infrared Hyperspectral Imaging and Chemometrics Algorithms

**DOI:** 10.1371/journal.pone.0098522

**Published:** 2014-05-30

**Authors:** Chuanqi Xie, Qiaonan Wang, Yong He

**Affiliations:** College of Biosystems Engineering and Food Science, Zhejiang University, Hangzhou, China; Institute of Psychology, Chinese Academy of Sciences, China

## Abstract

This study investigated the feasibility of using near infrared hyperspectral imaging (NIR-HSI) technique for non-destructive identification of sesame oil. Hyperspectral images of four varieties of sesame oil were obtained in the spectral region of 874–1734 nm. Reflectance values were extracted from each region of interest (ROI) of each sample. Competitive adaptive reweighted sampling (CARS), successive projections algorithm (SPA) and *x*-loading weights (*x*-LW) were carried out to identify the most significant wavelengths. Based on the sixty-four, seven and five wavelengths suggested by CARS, SPA and *x*-LW, respectively, two classified models (least squares-support vector machine, LS-SVM and linear discriminant analysis,LDA) were established. Among the established models, CARS-LS-SVM and CARS-LDA models performed well with the highest classification rate (100%) in both calibration and prediction sets. SPA-LS-SVM and SPA-LDA models obtained better results (95.59% and 98.53% of classification rate in prediction set) with only seven wavelengths (938, 1160, 1214, 1406, 1656, 1659 and 1663 nm). The *x*-LW-LS-SVM and *x*-LW-LDA models also obtained satisfactory results (>80% of classification rate in prediction set) with the only five wavelengths (921, 925, 995, 1453 and 1663 nm). The results showed that NIR-HSI technique could be used to identify the varieties of sesame oil rapidly and non-destructively, and CARS, SPA and *x*-LW were effective wavelengths selection methods.

## Introduction

Sesame oil, which contains high nutrient value such as unsaturated fatty acid and vitamin E, is welcome by many people [1]. It includes 43% oleic and linoleic each, 9% palmitic and 4% stearic fatty acids [2]. Eating sesame oil can control blood cholesterol level [3], prevent atherosclerosis [4] and reduce the risks of heart attack, arteriosclerosis and cancer [5]. The variety is one of the most important factors that strongly associated with the quality features of sesame oil. Different varieties own different levels of nutrient values. Because of the great economic benefit of sesame oil, some unscrupulous traders used low value of sesame oil or illegal cooking oil to pretend to be high value of edible oil in recent years. The counterfeit sesame oil has not only harmed the consumers' economy interests but also throw a threat to the people's health. Therefore, in order to guarantee and promote high quality sesame oil produced, the identification of the variety of sesame oil is extremely essential.

The most conventional method to discriminate varieties of oil is the physical-chemical technique. Although the obtained result by using this technique is accurate, it must be pointed out that there were limitations for the method. For example, it is time-consuming, inefficient and destructive, and it requires a professional highly trained and qualified. Moreover, it cannot be used in on-line identification, in the industry. Thus, an advanced method to identify the varieties of sesame oil is in urgent need.

**Figure 1:**
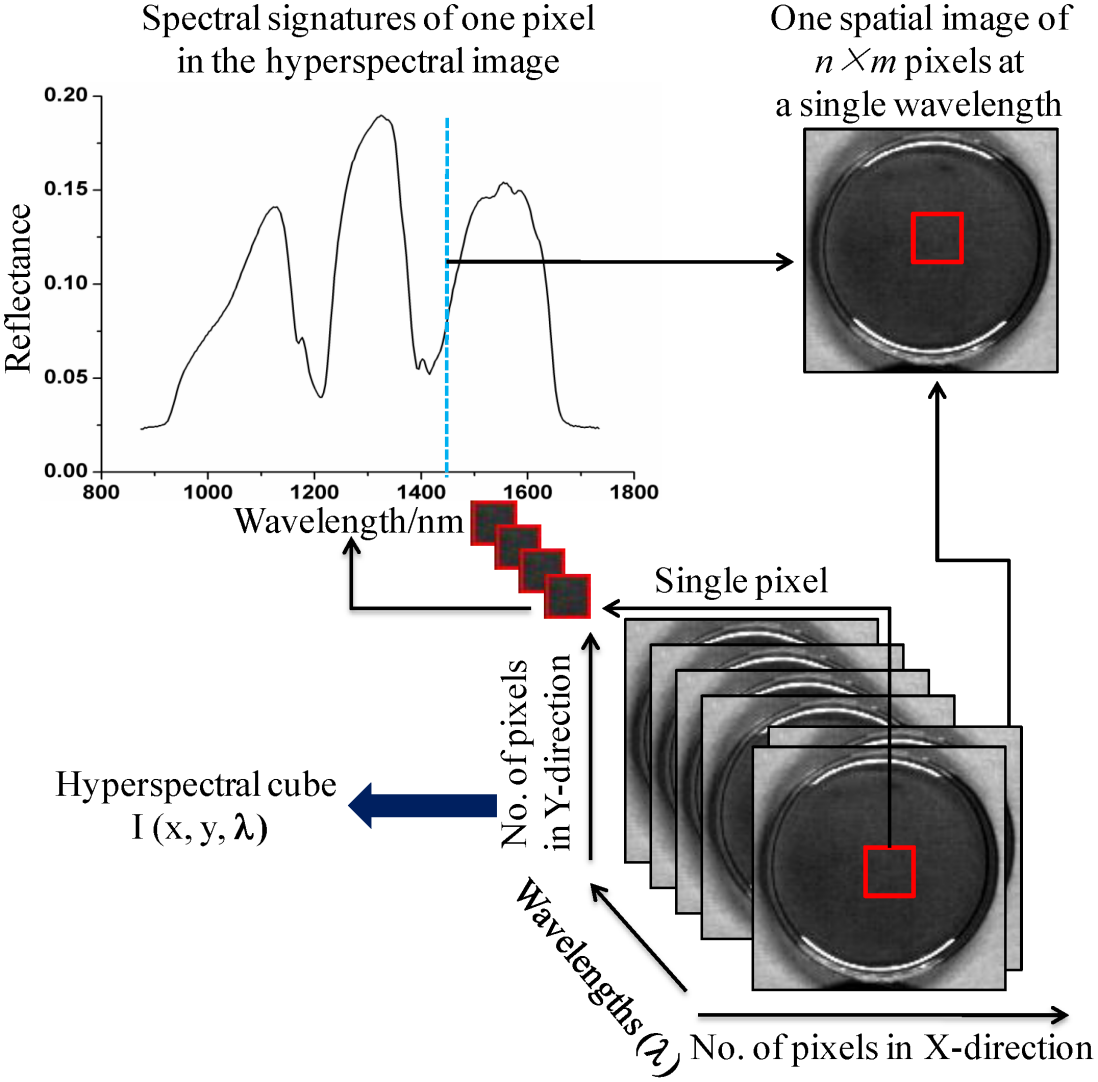
Hyperspectral imaging.

At present, spectral technique has been used for identification the oil [6], [7], [8]. Compared to the physical-chemical method mentioned above, spectral technique has many advantages such as fast, nondestructive, low cost and accurate. However, to identify the varieties of sesame oil is not usually found, especially using hyperspectral imaging technique. Near infrared hyperspectral imaging (NIR-HSI) integrates both spectral and imaging techniques together. NIR-HIS technique has already been widely studied in many fields due to its advantages [9], [10], [11]. By hyperspectral imaging system, one pixel of each hyperspectral image has a wavelength covering the whole spectral range. Finally, a spatial map (hyperspectral cube), which is composed of a series of images at each wavelength, is generated (Figure 1).

The aim of this study was carried out to develop a method to identify the varieties of sesame oil by using NIR-HSI technique based on spectral information. The objectives of this work were: (1) to find the quantitative relationships between the spectral information and the varieties of sesame oil; (2) to select effective wavelengths that are useful for the identification of varieties of sesame oil by CARS, SPA and *x*-LW, respectively; (3) to compare the performance of different identification models; (4) to identify the optimal calibration model for the identification of the varieties of sesame oil.

## Materials and Methods

### Flow of the study

The main steps of the whole procedures can be described as follows. Raw hyperspectral images of the four varieties of sesame oil were obtained by the NIR-HSI system across the wavelength region of 874–1734 nm in the first step. The raw hyperspectral images were corrected by equation (1), and the reflectance information of ROI of the corrected hyperspectral images was extracted to be treated as *X* variables. The samples were divided into calibration set and prediction set with the ratio of 2∶1. Identification models were then established based on full spectral wavelengths and selected wavelengths recommended by several effective variables selection algorithms (CARS, SPA and *x*-LW). Each selected wavelength suggested by SPA and *x*-LW was also used to establish identification model. Optimal identification model was selected by comparison in terms of the identification power (correct classification rate, CCR). Finally, identification of different varieties of sesame oil was achieved by the model.

### Hyperspectral imaging system and software

**Figure 2:**
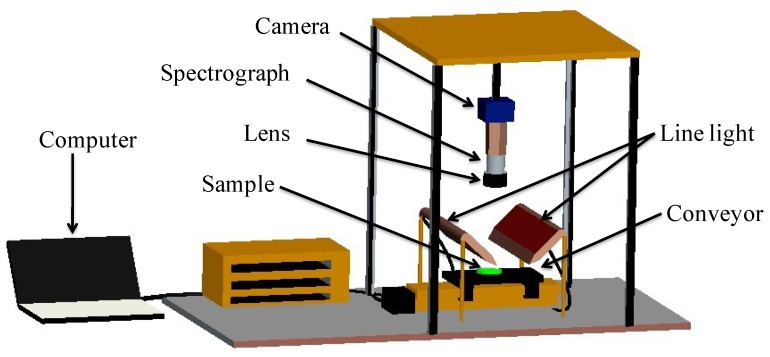
Schematic diagram of the NIR hyperspectral imaging system.

A near infrared hyperspectral imaging (NIR-HSI) system in the spectral range of 874–1734 nm was used as shown in Figure 2. The system contains a lens, an imaging spectrograph (N17E, Specim, Finland), a light source (Oriel Instruments, Irvine, Cal.) that included two 150 W quartz tungsten halogen lamps, a conveyor belt operated by a stepper motor (IRCP0076, Isuzu Optics Corp, Taiwan, China) and a computer. The area CCD array detector of the camera has 320×256 (spatial ×spectral) pixels, and the spectral resolution is 5 nm. The NIR-HSI system scans the sample line by line, and the reflected light was dispersed by the spectrograph and captured by the area CCD array detector in spatial-spectral(*x*×λ) axes. The ENVI 4.7 software (Research system Inc, Boulder, Co.USA), Unscrambler 9.7 software (Camo, Process, As, Oslo, Norway) and MATLAB R2009a (The Math Works, Natick, USA) software were used to preprocess the raw spectral information and establish identification models in this study.

### Samples

Four varieties of sesame oil including Huiyi, Liuyanghe, Taitaile and Xiaomo which been usually found in China, were purchased in the local market. Then, a volume of 60 ml of each variety of the sesame oil was evenly distributed in glass dishes of the same size (d = 90 mm). Each dish was then imaged individually by the NIR-HSI system. There were a total of 50 samples (50 glass dishes) of each variety.

### Image acquisition and correction

Each glass dish was placed on the conveyor belt to be scanned line by line by using the NIR-HSI system. The moving speed was set as 25 mm/sec and exposure time was 5 ms. Each hyperspectral image was obtained by using the imaging spectrograph of N17E across the wavelength region of 874–1734 nm. A raw hyperspectral image (hyperspectral cube) with a dimension of (*x, y, λ*) was created as the sample was scanned along the direction of the 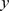
dimension. The dimension of the hyperspectral cube was 320 pixels in 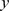
dimension and 256 bands in *λ* dimension. When the raw hyperspectral image was generated, it should be corrected into the reference hyperspectral image with black and white reference images based on the equation (1). The black reference image with the reflectance factor of 0% was obtained by turning off the light and keeping the lens being covered of its cap. The white one was obtained from a white Teflon board (CAL-tile200, 200 mm×25 mm×10 mm) with the reflectance factor of about 99%.
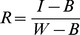
(1)Where *R* is the corrected hyperspectral image, *I* is the raw hyperspectral image, *B* is the black reference image, *W* is the white reference image.

### Data acquisition

An area with 25×25 pixels which was treated as the ROI (region of interest) was cropped from the center of each corrected hyperspectral image (each sample), resulting in a total of 200 samples of the four varieties of sesame oil. Reflectance values of all pixels of ROI were acquired by ENVI4.7 software. These spectral features were calculated via MATLAB R2009a software for establishing calibration model to identify different varieties of sesame oil.

A total of 33 samples were randomly picked out from each variety, which resulted in 132 samples of calibration set and 68 ones of prediction set [12]. The statistical information of each set was shown in Table 1.

**Table 1:** Statistical information of calibration and prediction sets.

Data sets	Huiyi	Liuyanghe	Taitaile	Xiaomo	Total
Calibration set	33	33	33	33	132
Prediction set	17	17	17	17	68
Total	50	50	50	50	200

### Calibration models

Least squares-support vector machine (LS-SVM), which has been widely used in many aspects [13], [14], can deal with both linear and nonlinear multivariate calibration problems [15]. A set of linear equations instead of a quadratic programming (QP) problem was applied in order to obtain the support vectors (SV) [16]. The radial basis function (RBF) was used in this study due to its excellent performance compared with other kernels. The LS-SVM algorithm could be described as follows:
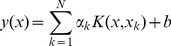
(2)


Where 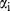 are Lagrange multipliers, 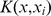 is the kernel function, 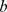 is the bias value.

The regularization parameter *gam (γ)* was used to determine the tradeoff between minimizing the training error and minimizing model complexity, and the width parameter *sig2 (σ^2^)* was used to defined the nonlinear mapping from input space to high-dimensional feature space [17]. The optimal parameter values of (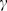, 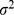) were calculated by grid search in this study. They were calculated by the free LS-SVM toolbox (LS-SVM v1.5, Suykens, Leuven, Belgium) in MATLAB R2009a.

Linear discriminant analysis (LDA) is a supervised recognition method used in statistics, pattern recognition and machine learning in order to find a linear combination of features that separate two or more classes of objects [18]. The principle of LDA for selection of latent variables is the maximum differentiation between the varieties and minimizes the variance within varieties. This algorithm produces a number of orthogonal linear discriminant functions, which allow the samples to be classified in one or another category [19].

### Effective wavelengths selection

The spectral information, which was acquired in the spectral region of 874 to 1734 nm, was characterized by high dimensionality with redundancy among contiguous wavelengths [20]. Therefore, the selection of effective wavelengths is a significant step in spectral studies [21]. The goal of effective wavelengths selection is to identify a subset of spectral features as smaller as possible to replace the full wavelengths for identification of different samples. The selected wavelengths can be equally or more efficient than the full spectral wavelengths [22]. Moreover, they cannot only reduce the dimensionality of raw data but also be used to develop the multispectral imaging identification system.

Competitive adaptive reweighted sampling (CARS) is an effective wavelengths selection algorithm. It selects effective wavelengths on the basis of the “survival of the fittest” principle. Firstly, it removes the wavelengths that are of small regression coefficients by exponentially decreasing function (EDF). Then, the ratio of wavelengths is calculated by an EDF equation [23]. The steps of each sampling run can be described as follows [24], [25]: (a) model sampling using Monte Carlo (MC) principle; (b) wavelengths selection based on EDF; (c) competitive wavelength selection by using adaptive reweighted sampling (ARS); (d) evaluation of the subset using cross validation. Finally, wavelengths that are of little or no effective information are eliminated while effective wavelengths are retained.

Successive projections algorithm (SPA), which aims to solve the collinear problems by selecting optimal variables with minimal redundancy, has been widely used in many fields [26], [27]. It uses a projection operation in a vector space for selecting key wavelengths with small collinearity [28].

In this study, *x*-loading weights (*x*-LW) were also used to select the most effective wavelengths for identification of varieties of sesame oil. It represents how much of each wavelength contributes to the variety variation in the data. The *x*-loading weights show how much of each wavelength contributes to explaining the response variation. Wavelengths with high loading weight values are significant for the varieties classification, and wavelengths with low loading weight values are not important [29]. Thus, the wavelengths with high absolute values of loading weight were considered as the key wavelengths while the low absolute values were rejected [30].

## Results and Discussion

### Spectral feature of tested samples

**Figure 3:**
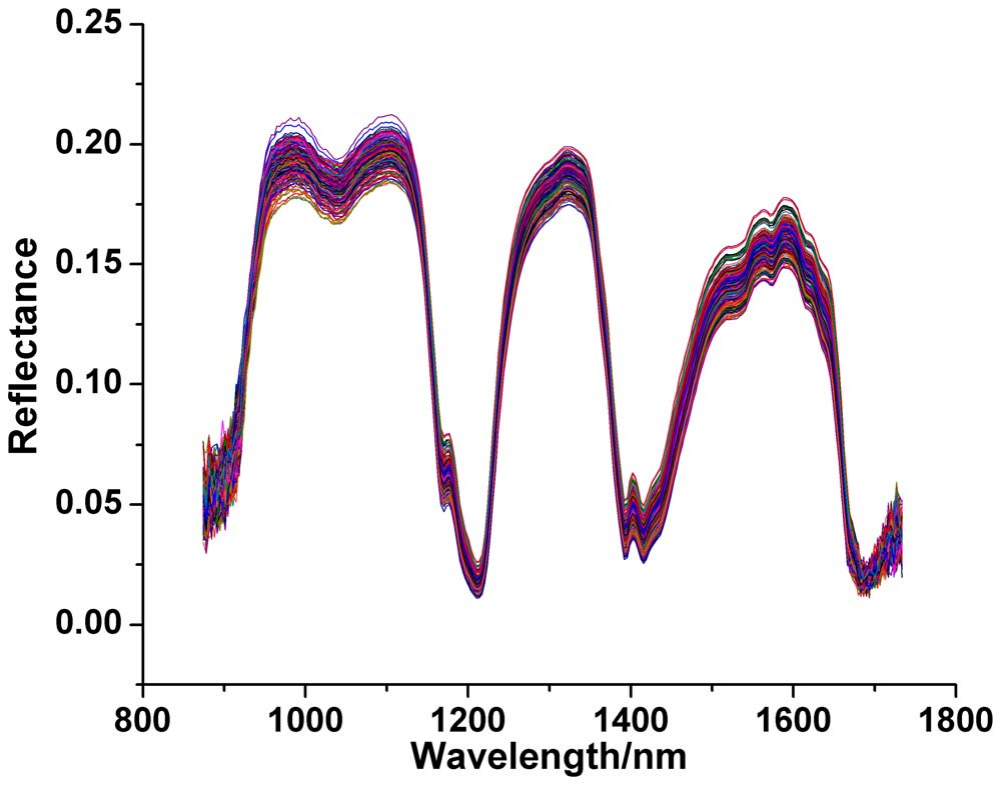
Spectral reflectance curves of the four different varieties of sesame oil.

The spectral reflectance curves of the four varieties of sesame oil were shown in Figure 3. Specifically, general trends of spectral curves of the four varieties of samples were similar with some spectral noise at the beginning and ending of the wavelengths. To eliminate noises and establish robust models, wavelengths at beginning and ending were rejected, resulting in spectral wavelengths from 921 to 1663 nm (bands 15 to 235) were used for further studies. Additionally, there were some strong absorption peaks, which were assigned to the functional groups such as C-H, C-C, C-N, C = O and O-H. However, there were no obvious differences among the spectral curves of the four varieties, which indicated that sesame oil could not be identified from spectral curves directly. In order to identify the varieties effectively, classification models based on chemometrics should be established.

### Identification model based on full wavelengths

In this study, identification model (LS-SVM) was first established based on full spectral wavelengths (821–1663 nm). The reflectance values extracted from ROI of hyperspectral image were treated as *X* variables, and the varieties were treated as *Y* variables (Huiyi-1, Liuyanghe-2, Taitaile-3, Xiaomo-4). The LS-SVM model obtained a satisfying result with the classification rate of 100% in the calibration set and 98.53% in prediction set. However, the input variables were too much, which will affect the robust and accurate of the discriminated model, increase the calculation time and could not be used in practical industry. Thus, several effective wavelengths selection methods were used to select key wavelengths for establishing simplified models.

### Effective wavelengths

#### Effective wavelengths recommended by CARS

**Figure 4:**
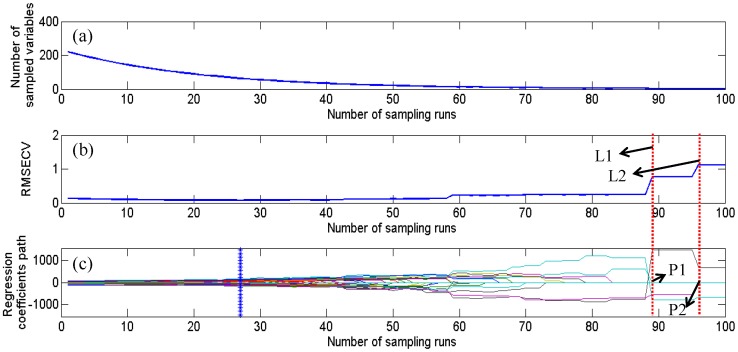
The changing trend of the number of sampled variables (a), 10-fold RMSECV values (b) and regression coefficients of each variable (c) with the increasing of sampling runs. The line (marked by asterisk).

In order to improve the performance of the identified ability and simplify the calibration model, CARS was firstly carried out to select effective wavelengths from the whole spectral wavelengths. It can be found in Figure 4 that the changing trend of the number of sampled variables (a), 10-fold *RMSECV* values (b) and the regression coefficient of each variable (c) with the increasing of sampling runs. In Figure 4 (a), it could be seen that the number of sampled variables decreased fast in the first step and slowly in the second step. In Figure 4 (b), the value of *RMSECV* firstly decreased which indicates the uninformative variables were eliminated, and then changed slightly which means variables do not change obviously, finally increased which is caused by the elimination of some key variables. In Figure 4 (c), each line represents the coefficient of each variable at different sampling runs. Some variables were extracted in each sampling run, and the optimal variables with the lowest value of *RMSECV* were marked by the vertical asterisk line. After the asterisk line, the value of *RMSECV* increased which owes to the removing of some effective wavelengths. The value of *RMSECV* sharply rose up to a higher stage at the point of dot line L1 because one variable (P1) dropped to zero. A same case is that a sharp rising of the value of *RMSECV* (L2) which was caused by another variable (P2) dropping to zero. In the CARS calculation, some variables were eliminated while some key variables were retained. As a result, sixty-four wavelengths were identified as the optimal wavelengths which were shown in Table 2. The number of selected variables was only 28.96% of that of the whole wavebands (Band15-Band235). These wavelengths were then used to replace the full wavelengths for identification of sesame oil. They were extremely relevant for the identification of sesame oil. The spectral data set was reduced to a matrix with a dimension of 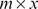, where 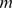
was the number of samples and 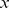
was the number of selected wavelengths.

**Table 2:** Effective wavelengths suggested by CARS.

Algorithm	Number	Selected wavelengths/nm
CARS	64	962, 975, 985, 999, 1012, 1046, 1049, 1052, 1056, 1076, 1109, 1113, 1130, 1143, 1167, 1170, 1193, 1197, 1200, 1207, 1214, 1220, 1230, 1234, 1274, 1288, 1291, 1301, 1311, 1321, 1325, 1342, 1345, 1352, 1359, 1375, 1382, 1396, 1399, 1402, 1406, 1413, 1419, 1429, 1433, 1500, 1507, 1517, 1521, 1541, 1544, 1551, 1554, 1565, 1588, 1601, 1605, 1632, 1639, 1642, 1649, 1652, 1656, 1659

#### Key wavelengths selected by SPA

**Figure 5:**
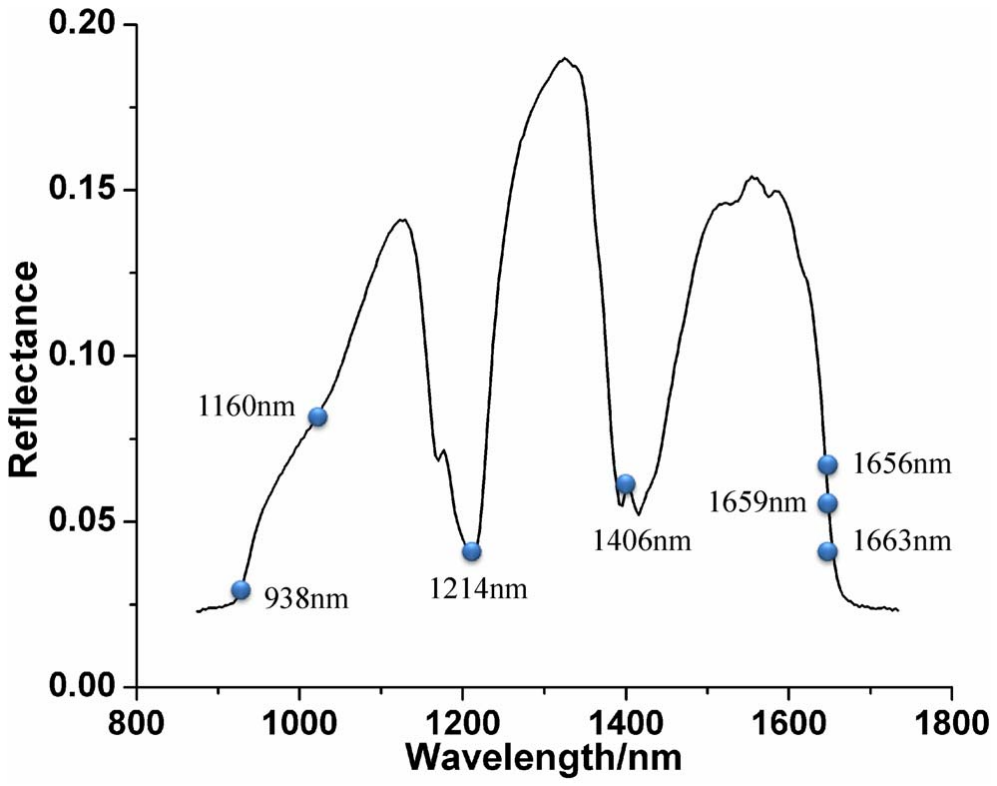
Effective wavelengths selected by SPA.

SPA algorithm was also carried out to select effective wavelengths from full wavelengths in this study. As a result, seven wavelengths (938, 1160, 1214, 1406, 1656, 1659 and 1663 nm) were identified as the optimal wavelengths which were shown in Figure 5. The selected wavelengths were used to replace the full wavelengths for identification of different varieties. The spectral dataset was reduced to a matrix with a dimension of 200×7 (200 was the number of samples and 7 was the number of selected wavelengths). Then, LS-SVM and LDA models based on the seven selected wavelengths was established.

#### Key wavelengths selected by *x*-LW

**Figure 6:**
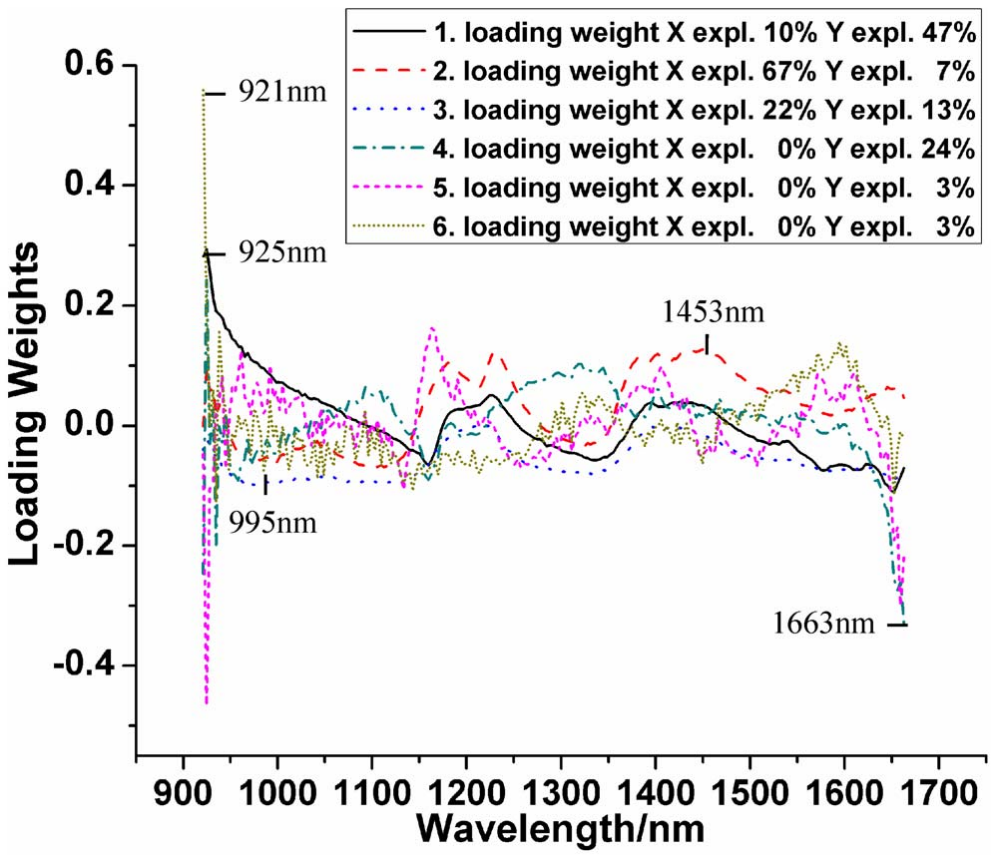
Effective wavelengths selected by *x*-LW.

Effective wavelengths for varieties classification were conducted based on *x*-loading weights. It can be seen in Figure 6 that the loading weights and explanation of *X* and *Y* variations. The number of loading weights was automatically determined by Unscrambler 9.7 software based on the minimum value of the predicted residual error sum of squares (PRESS) by full cross validation. The first six loading weights explained 99% of spectral variances and 97% of concentration variances, respectively. It suggested that the six loadings could be used to represent the full spectral wavelengths. Thus, wavelengths corresponding to the highest absolute values were selected as key wavelengths. The first and fifth loading obtained the same result (925 nm). Finally, a total of five wavelengths (921, 925, 995, 1453, and 1663 nm) were obtained. These wavelengths were then used to establish identification models.

### Identification models based on selected wavelengths

#### Identification models based on CARS

The LS-SVM and LDA models, which were established based on selected wavelengths suggested by CARS, obtained outstanding results with the CCR of 100% in both calibration and prediction sets. Compared with LS-SVM model, which were established based on full spectral wavelengths, there was a little increasing of the classification rate in CARS-LS-SVM model. The number of input variables of CARS-LS-SVM and CARS-LDA models was only 28.96% of that of the full spectral wavelengths. From the analysis, it could be found that CARS algorithm was an effective wavelengths selection method, and NIR-HIS could be used in the identification of varieties of sesame oil effectively. However, the number of input variables of CARS-LS-SVM and CARS-LDA models was a little more. Thus, other effective wavelengths selection methods should be used in the following analysis.

#### Identification model based on SPA

In this study, SPA was carried out to select effective variables. Then, SPA-LS-SVM and SPA-LDA models were established based on the selected wavelengths. The SPA-LS-SVM model obtained a satisfying result with the CCR of 100% in the calibration set and 95.59% in prediction set. The SPA-LDA model obtained an excellent result with the CCR of 100% in the calibration set and 98.53% in prediction set. Although, the CCR of SPA-LS-SVM model was a little decreasing compared with LS-SVM and CARS-LS-SVM models in prediction set, the number of its input variables was only seven, which was only account for 3.17% of that of LS-SVM model and 10.94% of that of CARS-LS-SVM model, respectively. Compared with CARS-LDA model, the CCR of SPA-LDA model was also a little decreasing in despite, while the number of its input variables was only seven. It was 3.17% of that of CARS-LDA model. The less variables suggested by SPA could not only simplify the model and speed up the calculated efficiency but also be used in practical industry. Thus, SPA was also an effective method to select key wavelengths.

#### Identification model based on *x*-LW

In this study, *x*-LW was also carried out to select effective variables. Then, *x*-LW-LS-SVM and *x*-LW-LDA models were established based on the selected wavelengths. Both of the two models performed well with the CCR greater than 80% in both calibration and prediction sets. Though the results obtained by *x*-LW-LS-SVM and *x*-LW-LDA models were a little worse than those obtained by the models established based on full spectral wavelengths, CARS and SPA, the results were acceptable and promising. However, the number of the input variables was only five, which was an account for 2.26% and 7.81% of those of full spectral wavelengths and wavelengths suggested by CARS. It demonstrated that *x*-LW was also an effective method to select key wavelengths.

### Classified result at each selected wavelength

**Figure 7:**
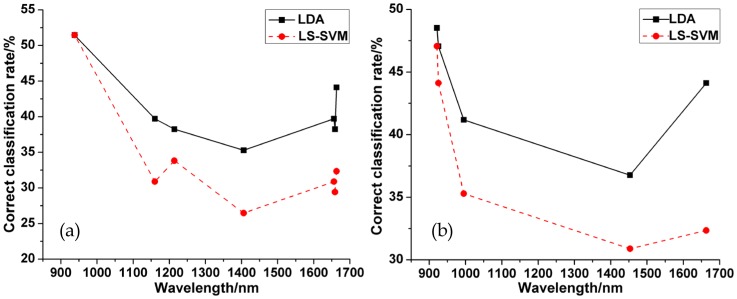
(a) Correct classification rate of each model at each selected wavelength suggested by SPA, (b) Correct classification rate of each model at each selected wavelength suggested by *x*-LW.

Each key wavelength, which was selected by SPA and *x*-LW algorithms, was also used to establish classification model. The results at each selected wavelength can be seen in Figure 7 (a) (b). It could be found that the classified results were different at different wavelengths. LDA model performed better than LS-SVM model at any wavelength. The general trend of CCRs firstly decreased and then increased in no matter LDA or LS-SVM models. In Figure 7 (a), the wavelength of 938 nm performed best with the CCR of 51.47% in both LDA and LS-SVM models. The wavelength of 1406 nm performed relatively worse with the CCR of 35.29% in LDA and 26.47% in LS-SVM models, respectively. In Figure 7 (b), the wavelength of 921 nm performed best with the CCR of 48.53% in LDA and 47.06% in LS-SVM models, respectively. The wavelength of 1453 nm performed relatively worse with the CCR of 36.76% in LDA and 30.88% in LS-SVM models, respectively. From above analysis, it can be seen that some wavelengths played prominent roles in the classification of varieties while some other wavelengths did not.

### Comparison of different models based on full wavelengths and selected wavelengths

The results of the seven identification models established based on full spectral wavelengths, and selected wavelengths (suggested by CARS, SPA and *x*-LW) were shown in Table 3. Different classification results, ranging from 82.35% to 100%, were shown in Table 3. From Table 3, it could be found that each model obtained an outstanding result. However, the number of full spectral wavebands was too much though the result was excellent (100% in the calibration set and 98.53% in prediction set). CARS-LS-SVM and CARS-LDA models performed better than LS-SVM model with a little increasing of CCR in prediction set and less input variables. It may because that the redundant information, which existed in the large number of input variables, affected the robust and ability of the model. The selected wavelengths contained most of the effective information and little redundant information. The number of input variables of SPA-LS-SVM and SPA-LDA models was only seven. It decreased largely compared to the full spectral wavelengths and wavelengths suggested by CARS. The obtained results were 95.59% and 98.53% in prediction sets of SPA-LS-SVM and SPA-LDA models, respectively. Though the CCRs obtained by *x*-LW-LS-SVM and *x*-LW-LDA models were lower than those obtained by other models, the results were acceptable. Both of the two models obtained the CCRs greater than 80%. More, the number of the input variables was only account for 2.26% of that of full spectral wavelengths. The results obtained by selected wavelengths were acceptable and encouraged for further study. The less input variables greatly accelerate the calculated speed and simplify the model. It demonstrates again that NIR-HSI technique could be used to identify the varieties of sesame oil, and CARS, SPA and *x*-LW were effective wavelengths selection methods.

**Table 3:** Correct classification rate of different models based on different wavelengths selection methods.

Number	Classification model	Number of wavelengths	Calibration			Prediction		
			No.	Missed	CCR^*^/%	No.	Missed	CCR^*^/%
1	LS-SVM	221	132	0	100	68	1	98.53
2	CARS-LS-SVM	64	132	0	100	68	0	100
3	CARS-LDA	64	132	0	100	68	0	100
4	SPA-LS-SVM	7	132	0	100	68	3	95.59
5	SPA-LDA	7	132	0	100	68	1	98.53
6	*x*-LW-LS-SVM	5	132	13	90.15	68	12	82.35
7	*x*-LW-LDA	5	132	18	86.36	68	9	86.76

CCR^*^ Correct Classification Rate.

## Conclusion

This study was carried out to evaluate the feasibility of using NIR-HSI system, which covers the spectral range of 874 to1734 nm, to identify the varieties of sesame oil. The overall results in this study indicated that NIR-HSI technique had the potential to be used to discriminate different varieties of sesame oil. CARS, SPA and *x*-LW were conducted to select effective wavelengths for establish identification model. Each model obtained an outstanding result with the CCR greater than 80%. CARS-LS-SVM and CARS-LDA models obtained the highest value of CCR of 100% with 64 input variables. The SPA-LS-SVM and SPA-LDA models obtained better results (95.59% and 98.53%) with only seven wavelengths. The *x*-LW-LS-SVM and *x*-LW-LDA models also obtained excellent results (>80% of CCR) with only five wavelengths. Among the wavelengths selected by SPA (38, 1160, 1214, 1406, 1656, 1659 and 1663 nm), wavelength of 938 nm performed best. The wavelength of 921 nm played the most prominent role among the wavelengths selected by *x*-LW (921, 925, 995, 1453 and 1663 nm). From the results, it could be seen that NIR-HSI technique could be used to identify the varieties of sesame oil rapidly and non-destructively, and CARS, SPA and *x*-LW were effective wavelengths selection methods.

However, this study was only a preliminary work. In future study, more samples with different varieties and more different spectral parameters should be used for establishing more robust and accurate model which could be used in practical industry.
